# The Association Between Thyroid Diseases and Alzheimer’s Disease in a National Health Screening Cohort in Korea

**DOI:** 10.3389/fendo.2022.815063

**Published:** 2022-03-07

**Authors:** Ji Hee Kim, Heui Seung Lee, Yoo Hwan Kim, Mi Jung Kwon, Joo-Hee Kim, Chan Yang Min, Dae Myoung Yoo, Hyo Geun Choi

**Affiliations:** ^1^ Department of Neurosurgery, Hallym University College of Medicine, Anyang, South Korea; ^2^ Department of Neurology, Hallym University College of Medicine, Anyang, South Korea; ^3^ Department of Pathology, Hallym University College of Medicine, Anyang, South Korea; ^4^ Division of Pulmonary, Allergy, and Critical Care Medicine, Department of Medicine, Hallym University College of Medicine, Anyang, South Korea; ^5^ Hallym Data Science Laboratory, Hallym University College of Medicine, Anyang, South Korea; ^6^ Department of Otorhinolaryngology-Head & Neck Surgery, Hallym University College of Medicine, Anyang, South Korea

**Keywords:** Alzheimer’s disease (AD), cognitive decline, neurodegeneration, neurodegenerative diseases, thyroid disease

## Abstract

**Objectives:**

Thyroid dysfunction is linked to an increased risk of cognitive impairment. However, studies on the relationships between thyroid diseases and Alzheimer’s disease (AD) have reported conflicting results. We investigated the associations between several thyroid diseases and AD in a nested case-control study.

**Methods:**

A total of 1,977 participants with AD were identified by claims data from 2002-2015 among a random sample of half a million people in the Korean National Health Insurance database. We recruited 16,473 age- and sex-matched (1:4 ratio) control participants and applied conditional logistic regression to estimate the relationships between thyroid diseases and AD, with adjustments for potential confounders, such as basic demographics, lifestyle factors, and various medical conditions or comorbidities.

**Results:**

The prevalence rates of hypothyroidism (odds ratio [OR]=1.14, 95% confidence interval [CI]=1.00-1.30), thyroiditis (OR=1.22, 95% CI=1.05-1.40), and hyperthyroidism (OR=1.13, 95% CI=1.01-1.28) were significantly higher in participants with AD than in control participants after adjustment for confounders.

**Conclusion:**

In this large national sample, we found significant relationships between several thyroid diseases and AD. Despite of the need for further investigation, these findings could better support to appreciate the pathophysiology of AD.

## Introduction

Thyroid hormones are essential for neuronal development and cellular metabolism, and their dysfunction can lead to potentially devastating health consequences that influence numerous organs in patients of all ages (1). In particular, thyroid hormones regulate neuronal cytoarchitecture, growth and synaptogenesis, and their receptors have a broad distribution in the central nervous system (CNS) (2). Accordingly, thyroid dysfunction, namely, any deficiency or increase in thyroid hormones, can induce a range of changes in mood and cognitive function and, in severe cases, cause anxiety, depression, irritability, and a deficit in executive function (3). Specifically, hypothyroidism can often be accompanied by widespread cognitive decline, particularly memory dysfunction, whereas overt thyrotoxicosis can manifest cognitive decline mainly in the areas of attention, concentration, and executive function (3).

Alzheimer’s disease (AD) is the most prevalent form of dementia and a progressive neurodegenerative disease of the CNS that leads to multidomain cognitive impairment, particularly memory dysfunction. According to the 2010 US Census data, the overall prevalence of AD was estimated to be 14.5% and the annual incidence was 2.3% (4). Pathologically, this disease is known to be attributed to extracellular amyloid deposition and intracellular neurofibrillary tangles of hyperphosphorylated tau (5). However, the causes of these features and those of the disease have not yet been elucidated.

After the link between clinical thyroid dysfunction and cognitive abnormalities was first described by Asher as “myxoedematous madness” in 1949 (6), interest in the association between thyroid disorders and dementia increased. Since neurotransmission, memory, and further vital brain functions require the maintenance of normal energy (glucose)-consuming processes, low thyroid function at any age can debilitate cognitive function (7). Several studies have also disclosed that thyroid hormones regulate the function of the adult brain, which illustrates the tightened arrangement of thyroid hormone transport into the brain, region-specific T4 (thyroxine) to T3 (tri-iodothyronine) conversion and T3 receptor levels (8). Given the theoretically increased risk of cognitive decline with thyroid dysfunction, it is conceivable that thyroid diseases can contribute to the pathophysiology of AD. Clinical observations and experimental studies have indicated a relationship between thyroid hormones and AD or its pathology (9–12). However, many previous studies provided inconsistent results owing to small sizes of the data samples, heterogeneous participant characteristics, or cognitive tests of limited sensitivity. Some studies have shown a positive association (12, 13), while others have reported no relationship between thyroid-stimulating hormone (TSH) or thyroid hormones and AD (14, 15).

Therefore, our hypothesis in this study is that thyroid dysfunction, whether clinical hypothyroidism or clinical hyperthyroidism, may be associated with AD in data from a large sample. To test this hypothesis, we aimed to investigate the associations between several thyroid diseases and AD using nationwide cohort data from Korea.

## Materials and Methods

### Study Population

The study protocol was approved by the ethics committee of Hallym University (2019-10-023). The data source for this nested-case control study was the Korean National Health Insurance Service (NHIS)-Health Screening Cohort, which included all the original claims data. Specific statements from the Korean NHIS-Health Screening Cohort data have been explained in greater detail in our previous report (16).

Participants who received treatment with levothyroxine for more than 3 months or who had goiter, hypothyroidism, thyroiditis, or hyperthyroidism were evaluated as described previously (17), and these conditions were included as independent variables. Participants with AD, which was used as the dependent variable in our analysis, were assessed and included as reported in our previous work (18).

### Participant Selection

**Figure 1:**
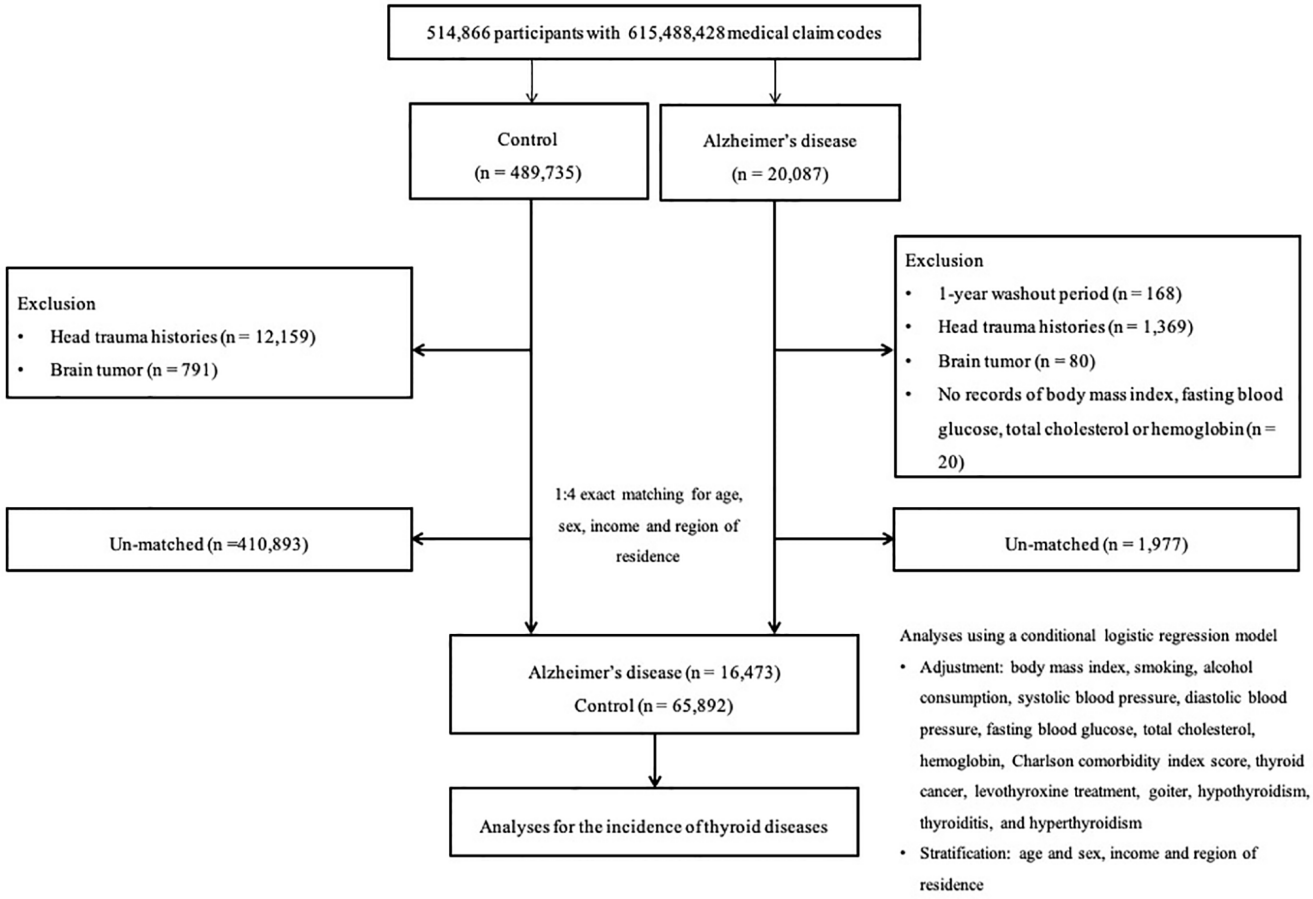
A schematic illustration of the participant selection process that was used in the present study. Of 514,866 participants, 16,473 Alzheimer’s disease participants were matched 1:4 with 65,892 control participants for age, sex, income, and region of residence.

AD participants were initially enrolled from a population of 514,866 participants with 615,488,428 medical claim codes between 2002 and 2015 (n=20,087). Participants were included as controls if they were not diagnosed with AD from 2002 to 2015 (n=489,735). Participants who were diagnosed with AD in 2002 were omitted from the study to select only participants who had been newly diagnosed with AD (n=168). In other words, the washout period was designated to exclude the participants who were continuously treated after being diagnosed with AD prior to 2002. To limit confounders, we excluded participants who had experienced head trauma (ICD-10 codes: S00 to S09, confirmed by neurologists, neurosurgeons, or emergency medicine doctors) ≥2 times (n=1,369 in AD; n=12,159 in control) and those who had been diagnosed with brain tumors (ICD-10 codes: C70-C72) at least two times (n=80 in AD; n=791 in control). AD participants who had no records of baseline body mass index (BMI), fasting blood glucose or total cholesterol and hemoglobin levels were also excluded (n=20). We matched AD participants and control participants at a 1:4 ratio based on participants’ age, sex, income, and region of residence. To shorten the selection bias, the control participants were assigned a random number order. The index date of each case was determined as the time of the first treatment for AD. Because the index date of control participants was defined as the index date of their corresponding AD participants, each matched AD and control participant had an identical index date. Throughout the matching step, 1,977 AD participants and 410,893 control participants were excluded. Eventually, 16,473 AD participants were matched with 65,892 control participants at a 1:4 ratio (**Figure 1**).

### Covariates

Our study covariates of interest included participant age, sex, income, region of residence, obesity, smoking, alcohol consumption, systolic blood pressure (SBP), diastolic blood pressure (DBS), fasting blood glucose, levels of total cholesterol and hemoglobin, and comorbidity score. Age was stratified into ten groups with intervals of 5 years: from 40-44 to 85+ years old group. Income groups were categorized into 5 classes from class 1 (lowest income) to class 5 (highest income), and each class comprised of 5 self-employment health insurance classes and 5 employment health insurance classes. The one health aid class was included in the lowest income class. The region of residence was categorized as urban or rural (19). Obesity assessments *via* BMI (kg/m^2^) was made in accordance with the Asia-Pacific criteria (20). Missing BMI was substituted by the mean values of variables from the selected people. Assessments of smoking status and alcohol consumption were performed as defined in a previous publication (16). The Charlson Comorbidity Index (CCI) was utilized to assess comorbidity load with a score from 0 to 29, excluding dementia, cancer, and metastatic cancer.

### Statistical Analysis

The balance of baseline characteristics between study groups was assessed by reporting standardized differences. To compare thyroid diseases between AD participants and control participants, odds ratios (ORs) and 95% confidence intervals (CIs) were computed by using a conditional logistic regression method. We further adjusted the ORs for possible covariates using an adjusted model. The covariates BMI, smoking, alcohol consumption, SBP CCI scores and thyroid cancer were adjusted for in model 1. Levothyroxine treatment, goiter, hypothyroidism, thyroiditis, and hyperthyroidism were additionally included in model 1 to estimate model 2. This is because levothyroxine treatment, goiter, hypothyroidism, thyroiditis, and hyperthyroidism histories were significantly linked to each other (each P <0.001, **Table S1**).

Subgroup analyses were performed to identify the incidence of thyroid diseases in terms of age and sex (over 75 years of age or under; men or women) and income and region of residence (low or high; urban or rural). Each subgroup was stratified into four groups, and the estimates were conducted using the unadjusted model, model 1, and model 2.

Two-tailed analyses were conducted, and P values less than 0.05 were considered to indicate a statistically significant difference. A standardized difference of ≤0.1 was deemed an ideal balance, and a standardized difference of ≤0.2 was treated as acceptable balance. Statistical analysis was conducted by using SAS ver. 9.4 (SAS Institute Inc., Cary, NC, USA).

## Results

### Characteristics of Participants

The baseline characteristics of the participants in this study are shown in **Table 1**. The study groups corresponded to age, sex, income, and region of residence (each standardized difference = 0.00). The proportions of other baseline characteristics and thyroid diseases were well balanced between the two groups, and the standardized differences were less than 0.2. However, the AD group tended to have slightly higher percentages of participants with levothyroxine treatment over 3 months, goiter, hypothyroidism, thyroiditis, and hyperthyroidism (**Table 1**).

**Table 1:** Baseline characteristics of the study population.

Characteristics	Total participants			
	Alzheimer’s disease patients (n, %)		Controls (n, %)	Standardized Difference
Total number	16,473 (100.0)		65,892 (100.0)	
Age (years)				0.00
40-44	1 (0.0)		4 (0.0)	
45-49	48 (0.3)		192 (0.3)	
50-54	171 (1.0)		684 (1.0)	
55-59	396 (2.4)		1,584 (2.4)	
60-64	903 (5.5)		3,612 (5.5)	
65-69	2,075 (12.6)		8,300 (12.6)	
70-74	3,992 (24.2)		15,968 (24.2)	
75-79	4,959 (30.1)		19,836 (30.1)	
80-84	3,419 (20.8)		13,676 (20.8)	
85+	509 (3.1)		2,036 (3.1)	
Sex				0.00
Male	6,406 (38.9)		25,624 (38.9)	
Female	10,067 (61.1)		40,268 (61.1)	
Income				0.00
1 (lowest)	3,375 (20.5)		13,500 (20.5)	
2	1,861 (11.3)		7,444 (11.3)	
3	2,259 (13.7)		9,036 (13.7)	
4	2,988 (18.1)		11,952 (18.1)	
5 (highest)	5,990 (36.4)		23,960 (36.4)	
Region of residence				0.00
Urban	5,821 (35.3)		23,284 (35.3)	
Rural	10,652 (64.7)		42,608 (64.7)	
Obesity^†^				0.11
Underweight	892 (5.4)		2,735 (4.2)	
Normal	6,614 (40.2)		23,997 (36.4)	
Overweight	3,859 (23.4)		16,710 (25.4)	
Obese I	4,620 (28.1)		20,358 (30.9)	
Obese II	488 (3.0)		2,092 (3.2)	
Smoking status				0.03
Nonsmoker	12,987 (78.8)		52,799 (80.1)	
Past smoker	1,059 (6.4)		3,992 (6.1)	
Current smoker	2,427 (14.7)		9,101 (13.8)	
Alcohol consumption				0.00
<1 time a week	13,558 (82.3)		54,218 (82.3)	
≥1 time a week	2,915 (17.7)		11,674 (17.7)	
Systolic blood pressure				0.03
<120 mmHg	3,700 (22.5)		14,081 (21.4)	
120-139 mmHg	7,792 (47.3)		31,939 (48.5)	
≥140 mmHg	4,981 (30.2)		19,872 (30.2)	
Diastolic blood pressure				0.03
<80 mmHg	7,290 (44.3)		29,635 (45.0)	
80-89 mmHg	5,892 (35.8)		23,738 (36.0)	
≥90 mmHg	3,291 (20.0)		12,519 (19.0)	
Fasting blood glucose				0.12
<100 mg/dL	8,960 (54.4)		38,217 (58.0)	
100-125 mg/dL	5,059 (30.7)		20,516 (31.1)	
≥126 mg/dL	2,454 (14.9)		7,159 (10.9)	
Total cholesterol				0.04
<200 mg/dL	8,912 (54.1)		35,858 (54.4)	
200-239 mg/dL	5,104 (31.0)		21,024 (31.9)	
≥240 mg/dL	2,457 (14.9)		9,010 (13.7)	
Hemoglobin (g/dL)				0.05
≥12 for men and ≥10 for women	15,765 (95.7)		63,689 (96.7)	
<12 for men and <10 for women	708 (4.3)		2,203 (3.3)	
CCI score^‡^				0.49
0	6,711 (40.7)		41,304 (62.7)	
1	4,449 (27.0)		14,570 (22.1)	
≥2	5,313 (32.3)		10,018 (15.2)	
Thyroid cancer	121 (0.7)		620 (0.9)	0.02
Period of levothyroxine treatment				0.02
<3 months	15,874 (96.4)		63,716 (96.7)	
≥3 months	599 (3.6)		2,176 (3.3)	
Goiter	591 (3.6)		2,239 (3.4)	0.01
Hypothyroidism	602 (3.7)		2,097 (3.2)	0.03
Thyroiditis	280 (1.7)		902 (1.4)	0.03
Hyperthyroidism	400 (2.4)		1,349 (2.1)	0.03

CCI, Charlson comorbidity index.

^†^Obesity (BMI, body mass index, kg/m^2^) was categorized as <18.5 (underweight), ≥18.5 to <23 (normal), ≥23 to <25 (overweight), ≥25 to <30 (obese I), and ≥30 (obese II).

^‡^CCI scores were calculated excluding dementia, cancer and metastatic cancer.

### Association Between Thyroid Diseases and AD

**Table 2** exhibits the logistic regression results of the analysis of the association between thyroid diseases and AD. Compared with no thyroid disease, all thyroid diseases were significantly associated with AD in multiple logistic regression model 1, which included all the covariates of interest. Among them, hypothyroidism (OR=1.14, 95% CI=1.00-1.30, P=0.046), thyroiditis (OR=1.22, 95% CI=1.05-1.40, P=0.008), and hyperthyroidism (OR=1.13, 95% CI=1.01-1.28, P=0.039) were still identified to have significant associations with AD in multiple logistic regression model 2 with each thyroid disease or condition being controlled.

**Table 2:** Crude and adjusted odds ratios (95% confidence intervals) associated with levothyroxine treatment, goiter, hypothyroidism, thyroiditis, and hyperthyroidism in Alzheimer’s disease patients compared to control participants.

Characteristics	N of Thyroid disease patients	N of Controls	Odds ratios for Alzheimer’s disease					
	(exposure/total, %)	(exposure/total, %)	Crude^†^	P-value	Model 1^†‡^	P-value	Model 2^†§^	P-value
Total participants (n = 82,365)								
Levothyroxine	599/16,473 (3.6%)	2,176/65,892 (3.3%)	1.11 (1.01-1.21)	0.033^*^	1.15 (1.04-1.27)	0.007^*^	0.98 (0.85-1.12)	0.738
Goiter	591/16,473 (3.6%)	2,239/65,892 (3.4%)	1.06 (0.97-1.16)	0.229	1.13 (1.03-1.25)	0.012^*^	1.08 (0.97-1.19)	0.154
Hypothyroidism	602/16,473 (3.7%)	2,097/65,892 (3.2%)	1.16 (1.05-1.27)	0.002^*^	1.19 (1.08-1.31)	0.001^*^	1.14 (1.00-1.30)	0.046^*^
Thyroiditis	280/16,473 (1.7%)	902/65,892 (1.4%)	1.25 (1.09-1.43)	0.001^*^	1.29 (1.12-1.48)	<0.001^*^	1.22 (1.05-1.40)	0.008^*^
Hyperthyroidism	400/16,473 (2.4%)	1,349/65,892 (2.0%)	1.19 (1.06-1.33)	0.002^*^	1.19 (1.06-1.33)	0.004^*^	1.13 (1.01-1.28)	0.039^*^

CCI, Charlson comorbidity index.

^*^Conditional logistic regression model, Significance at P < 0.05.

^†^Models stratified by age, sex, income, and region of residence.

^‡^Model 1 was adjusted for obesity, smoking, alcohol consumption, systolic blood pressure, diastolic blood pressure, fasting blood glucose, total cholesterol, hemoglobin, thyroid cancer, and CCI score.

^§^Model 2 was adjusted for the factors in Model 1 plus levothyroxine treatment, goiter, hypothyroidism, thyroiditis, and hyperthyroidism.

### Subgroup Analyses Stratified by Age, Sex, Income, and Region of Residence

We performed subgroup analyses to assess the effect of thyroid diseases on AD within subgroups stratified by age and sex, as indicated in **Table 3**. Within each subgroup, hypothyroidism, thyroiditis, and hyperthyroidism were consistently associated with a higher likelihood of having AD. Among these subgroups, only the group of men older than 75 years was statistically significant. As displayed in **Table 4**, the results from the subgroup stratified by income and region of residence indicated that hypothyroidism, thyroiditis, and hyperthyroidism were consistently linked to a higher likelihood of AD in all subgroups except for the subgroup of rural residents with low income.

**Table 3:** Subgroup analyses of crude and adjusted odds ratios (95% confidence intervals) associated with levothyroxine treatment, goiter, hypothyroidism, thyroiditis, and hyperthyroidism in Alzheimer’s disease patients compared to control participants stratified by age and sex.

Characteristics	N of Thyroid disease patients		N of Controls		Odds ratios for Alzheimer’s disease						
	(exposure/total, %)		(exposure/total, %)		Crude^†^		P-value	Model 1^†‡^	P-value	Model 2^†§^	P-value
Age <75 years, men (n = 14,950)											
Levothyroxine	52/2,990 (1.7%)		166/11,960 (1.4%)		1.26 (0.92-1.72)		0.153	1.32 (0.93-1.86)	0.124	1.05 (0.66-1.68)	0.831
Goiter	43/2,990 (1.4%)		205/11,960 (1.7%)		0.84 (0.60-1.17)		0.291	0.96 (0.68-1.36)	0.835	0.88 (0.61-1.25)	0.473
Hypothyroidism	59/2,990 (2.0%)		171/11,960 (1.4%)		1.39 (1.03-1.87)		0.032^*^	1.38 (1.00-1.90)	0.050^*^	1.29 (0.84-1.97)	0.247
Thyroiditis	23/2,990 (0.8%)		64/11,960 (0.5%)		1.44 (0.89-2.33)		0.134	1.59 (0.97-2.61)	0.065	1.50 (0.91-2.48)	0.115
Hyperthyroidism	45/2,990 (1.5%)		143/11,960 (1.2%)		1.26 (0.90-1.77)		0.176	1.29 (0.91-1.83)	0.155	1.20 (0.84-1.73)	0.322
Age ≥75 years old, men (n = 17,080)											
Levothyroxine	56/3,416 (1.6%)		195/13,664 (1.4%)		1.15 (0.85-1.55)		0.357	1.17 (0.85-1.60)	0.337	0.94 (0.62-1.42)	0.757
Goiter	42/3,416 (1.2%)		165/13,664 (1.2%)		1.02 (0.72-1.43)		0.916	1.05 (0.74-1.49)	0.777	0.99 (0.69-1.41)	0.934
Hypothyroidism	64/3,416 (1.9%)		207/13,664 (1.5%)		1.24 (0.94-1.65)		0.004^*^	1.25 (0.94-1.68)	0.003^*^	1.23 (0.84-1.80)	0.010^*^
Thyroiditis	41/3,416 (1.2%)		106/13,664 (0.8%)		1.55 (1.08-2.23)		0.017^*^	1.63 (1.13-2.36)	0.009^*^	1.56 (1.07-2.26)	0.020^*^
Hyperthyroidism	50/3,416 (1.5%)		129/13,664 (0.9%)		1.56 (1.12-2.17)		0.008^*^	1.55 (1.11-2.17)	0.011^*^	1.49 (1.06-2.09)	0.023^*^
Age <75 years, women (n = 22,980)											
Levothyroxine	274/4,596 (6.0%)		1,002/18,384 (5.5%)		1.10 (0.96-1.26)		0.176	1.16 (1.00-1.35)	0.056	0.98 (0.80-1.21)	0.870
Goiter	330/4,596 (7.2%)		1,128/18,384 (6.1%)		1.18 (1.04-1.35)		0.009^*^	1.28 (1.12-1.46)	<0.001^*^	1.24 (1.08-1.43)	0.003^*^
Hypothyroidism	261/4,596 (5.7%)		951/18,384 (5.2%)		1.10 (0.96-1.27)		0.170	1.16 (1.00-1.34)	0.056	1.09 (0.90-1.32)	0.389
Thyroiditis	114/4,596 (2.5%)		398/18,384 (2.2%)		1.15 (0.93-1.42)		0.195	1.20 (0.97-1.49)	0.100	1.09 (0.87-1.37)	0.444
Hyperthyroidism	163/4,596 (3.5%)		575/18,384 (3.1%)		1.14 (0.95-1.36)		0.150	1.13 (0.94-1.35)	0.207	1.06 (0.88-1.28)	0.524
Age ≥75 years old, women (n = 27,355)											
Levothyroxine	217/5,471 (4.0%)		813/21,884 (3.7%)		1.07 (0.92-1.25)		0.382	1.11 (0.94-1.31)	0.203	0.97 (0.77-1.23)	0.825
Goiter	176/5,471 (3.2%)		741/21,884 (3.4%)		0.95 (0.80-1.12)		0.554	1.00 (0.84-1.19)	0.970	0.95 (0.79-1.14)	0.591
Hypothyroidism	218/5,471 (4.0%)		768/21,884 (3.5%)		1.14 (0.98-1.33)		0.092	1.18 (1.00-1.38)	0.044^*^	1.17 (0.94-1.46)	0.155
Thyroiditis	102/5,471 (1.9%)		334/21,884 (1.5%)		1.23 (0.98-1.53)		0.075	1.24 (0.98-1.55)	0.068	1.20 (0.95-1.51)	0.132
Hyperthyroidism	142/5,471 (2.6%)		502/21,884 (2.3%)		1.14 (0.94-1.37)		0.188	1.14 (0.94-1.38)	0.190	1.10 (0.90-1.34)	0.340

CCI, Charlson comorbidity index.

^*^Conditional logistic regression model, Significance at P < 0.05.

^†^Models stratified by age, sex, income, and region of residence.

^‡^Model 1 was adjusted for obesity, smoking, alcohol consumption, systolic blood pressure, diastolic blood pressure, fasting blood glucose, total cholesterol, hemoglobin, thyroid cancer, and CCI score.

^§^Model 2 was adjusted for the factors in Model 1 plus levothyroxine treatment, goiter, hypothyroidism, thyroiditis, and hyperthyroidism.

**Table 4:** Subgroup analyses of crude and adjusted odds ratios (95% confidence intervals) associated with levothyroxine treatment, goiter, hypothyroidism, thyroiditis, and hyperthyroidism in Alzheimer’s disease patients compared to control participants stratified by income and region of residence.

Characteristics	N of Thyroid disease patients		N of Controls		Odds ratios for Alzheimer’s disease						
	(exposure/total, %)		(exposure/total, %)		Crude^†^		P-value	Model 1^†‡^	P-value	Model 2^†§^	P-value
Low income, urban (n = 11,730)											
Levothyroxine	87/2,346 (3.7%)		350/9,384 (3.7%)		0.99 (0.78-1.26)		0.961	0.99 (0.76-1.29)	0.927	0.85 (0.59-1.22)	0.374
Goiter	86/2,346 (3.7%)		350/9,384 (3.7%)		0.98 (0.77-1.25)		0.883	1.06 (0.83-1.37)	0.639	1.03 (0.79-1.35)	0.806
Hypothyroidism	89/2,346 (3.8%)		329/9,384 (3.5%)		1.09 (0.86-1.38)		0.500	1.07 (0.83-1.38)	0.600	1.12 (0.80-1.56)	0.507
Thyroiditis	45/2,346 (1.9%)		141/9,384 (1.5%)		1.28 (0.91-1.80)		0.150	1.36 (0.96-1.93)	0.086	1.37 (0.95-1.97)	0.097
Hyperthyroidism	53/2,346 (2.3%)		193/9,384 (2.1%)		1.10 (0.81-1.50)		0.540	1.10 (0.80-1.51)	0.570	1.08 (0.78-1.50)	0.643
Low income, rural (n = 25,745)											
Levothyroxine	163/5,149 (3.2%)		530/20,596 (2.6%)		1.24 (1.04-1.48)		0.019^*^	1.26 (1.04-1.53)	0.019^*^	0.98 (0.74-1.28)	0.858
Goiter	144/5,149 (2.8%)		529/20,596 (2.6%)		1.09 (0.91-1.32)		0.356	1.17 (0.96-1.42)	0.111	1.08 (0.88-1.33)	0.459
Hypothyroidism	171/5,149 (3.3%)		506/20,596 (2.5%)		1.37 (1.15-1.63)		0.001^*^	1.38 (1.14-1.66)	0.001^*^	1.34 (1.05-1.72)	0.021^*^
Thyroiditis	79/5,149 (1.5%)		240/20,596 (1.2%)		1.32 (1.02-1.71)		0.033^*^	1.39 (1.07-1.80)	0.015^*^	1.29 (0.98-1.69)	0.065
Hyperthyroidism	99/5,149 (1.9%)		378/20,596 (1.8%)		1.05 (0.84-1.31)		0.677	1.07 (0.85-1.35)	0.563	0.98 (0.78-1.24)	0.882
High income, urban (n = 17,375)											
Levothyroxine	160/3,475 (4.6%)		567/13,900 (4.1%)		1.14 (0.95-1.36)		0.165	1.23 (1.01-1.50)	0.037^*^	1.09 (0.83-1.43)	0.523
Goiter	163/3,475 (4.7%)		610/13,900 (4.4%)		1.08 (0.90-1.28)		0.428	1.15 (0.96-1.39)	0.130	1.08 (0.89-1.31)	0.444
Hypothyroidism	163/3,475 (4.7%)		572/13,900 (4.1%)		1.15 (0.96-1.37)		0.130	1.19 (0.99-1.44)	0.064	1.07 (0.84-1.37)	0.587
Thyroiditis	65/3,475 (1.9%)		213/13,900 (1.5%)		1.23 (0.93-1.62)		0.155	1.28 (0.96-1.71)	0.089	1.18 (0.88-1.58)	0.278
Hyperthyroidism	104/3,475 (3.0%)		326/13,900 (2.3%)		1.29 (1.03-1.61)		0.028^*^	1.25 (0.99-1.57)	0.061	1.17 (0.93-1.49)	0.186
High income, rural (n = 27,515)											
Levothyroxine	189/5,503 (3.4%)		729/22,012 (3.3%)		1.04 (0.88-1.22)		0.645	1.09 (0.91-1.30)	0.340	0.96 (0.75-1.23)	0.753
Goiter	198/5,503 (3.6%)		750/22,012 (3.4%)		1.06 (0.90-1.24)		0.478	1.13 (0.96-1.33)	0.158	1.09 (0.91-1.30)	0.340
Hypothyroidism	179/5,503 (3.3%)		690/22,012 (3.1%)		1.04 (0.88-1.23)		0.653	1.10 (0.92-1.31)	0.296	1.06 (0.84-1.33)	0.638
Thyroiditis	91/5,503 (1.7%)		308/22,012 (1.4%)		1.19 (0.94-1.50)		0.158	1.18 (0.93-1.51)	0.172	1.13 (0.88-1.45)	0.324
Hyperthyroidism	144/5,503 (2.6%)		452/22,012 (2.1%)		1.28 (1.06-1.55)		0.010^*^	1.29 (1.06-1.56)	0.011^*^	1.26 (1.03-1.53)	0.025^*^

CCI, Charlson comorbidity index.

^*^Conditional logistic regression model, Significance at P < 0.05.

^†^Models stratified by age, sex, income, and region of residence.

^‡^Model 1 was adjusted for obesity, smoking, alcohol consumption, systolic blood pressure, diastolic blood pressure, fasting blood glucose, total cholesterol, hemoglobin, thyroid cancer, and CCI score.

^§^Model 2 was adjusted for the factors in Model 1 plus levothyroxine treatment, goiter, hypothyroidism, thyroiditis, and hyperthyroidism.

## Discussion

This study demonstrated the associations between hypothyroidism, thyroiditis, hyperthyroidism, and AD, with OR values of 1.14, 1.22, and 1.13 after adjusting the model for covariates and each thyroid disease or condition. These findings are consistent with epidemiological studies revealing the effect of clinical hypothyroidism and clinical hyperthyroidism on the risk of dementia in large sample sizes (13, 21–23). George et al. showed that overt hyperthyroidism was related to a higher risk of dementia than euthyroidism in a prospective cohort study of community-dwelling adults who were followed for 22 years (hazard ratio [HR] = 1.40). The authors highlighted the importance of diagnosing and treating hyperthyroidism (22). Forlkestad et al. found an increased risk of dementia in hyperthyroid individuals in two patient cohorts using large-scale registry-based data (HR=1.17 and 1.06). In addition, the researchers revealed that every 6 months of decreased TSH was correlated with a 16% increased possibility of dementia compared to that of people with normal TSH, suggesting that the longer a patient has hyperthyroidism, the greater the risk of developing dementia is (21). According to a Framingham study by Tan et al. that analyzed prospectively collected data, there was a strong association between hypothyroidism and the risk of Alzheimer-type dementia, specifying that overt thyroid dysfunction translated to a 2-fold greater risk for the development of Alzheimer-type dementia than euthyroidism (13). In the SADEM study, investigators demonstrated that the relationship of thyroid dysfunction with cognitive decline was most obvious in overt hypothyroidism (OR=1.23) (23).

As with many other studies, including the complex interplay between hormonal axes, contradictory evidence is often also found for these associations. In a population-based cohort study of 1,077 elderly people in the Netherlands, the author reported no association between TSH/thyroid hormones and the risk of dementia (HR=1.00) or AD (HR=0.97) (9). The fact that the number of dementia patients with high or low TSH was relatively small restricted the power of the result, and together with the short follow-up, this can lead to decreased precision. Formigia et al. reported that subclinical hypothyroidism and hyperthyroidism were not related to a higher risk of cognitive function in their population-based, prospective cohort study consisting of 307 inhabitants (24). They explained that the main limitation of their study was the small number of individuals with abnormal TSH values. Additionally, a recent meta-analysis showed that except for subclinical hyperthyroidism, clinical hyperthyroidism and clinical hyperthyroidism did not influence dementia. The difference between the impact of clinical or subclinical hyperthyroidism on dementia can be clarified by the shortage of treatment and prolonged period of thyroid dysregulation in patients with subclinical hyperthyroidism. However, the authors identified adverse effects of both low and high TSH concentrations in the dose-response meta-analytic study, which inferred that not only hyperthyroidism but also hypothyroidism could cause dementia (25).

Notably, our findings also demonstrated that there was a significant association with thyroiditis and AD. To date, no study has addressed the link between all-cause thyroiditis and dementia and AD. It has been mainly supposed that an increased risk of cognitive dysfunction could pertain to autoimmune disease rather than thyroiditis per se, since the most common cause of thyroiditis is an autoimmune disease. In addition, an increased risk for dementia and AD was noted in patients with Hashimoto’s thyroiditis/hypothyroidism in a previous study, which has been suggested to be due to thyroid dysfunction (13, 26). Although our analysis could not consider the various etiologies of thyroiditis, such as autoimmunity, infection, or drugs, our data confirmed that the relationship between thyroiditis and AD was still statistically significant when controlling for each thyroid disease or condition as well as various comorbidities.

Proposed neuropathologic mechanisms contributing to the association of thyroid dysfunction with AD have been established in the probable roles of endogenous thyroid hormones and thyrotropin-releasing hormone (TRH). The first potential mechanism is amyloid plaque accumulation. This is due to increased amyloid precursor protein gene expression by low CNS thyroid hormone levels, as thyroid hormones are involved in the regulation of amyloid beta and amyloid beta precursor genes (11). The second potential mechanism is excessive thyroid hormone levels, which have been associated with toxic effects, such as increased oxidative stress on neuronal viability and enhanced neuronal death, which additionally increase vulnerability of the brain to amyloid toxicity (11, 27, 28). The third potential mechanism is the direct adverse effect of thyroxine (T4) reduction on cholinergic neurons. Several experimental studies have indicated the significant function of thyroid hormones in the development and preservation of the basal forebrain cholinergic neurons involved in AD (29, 30). The fourth potential mechanism is the elucidation of the relationship between TRH and the phosphorylation of tau protein. A number of studies have confirmed that a decrease in TRH could improve the phosphorylation of tau and other proteins, which are theoretically involved in the pathogenesis of AD (10, 31). The fifth potential mechanism is the induction of acetylcholine synthesis and release by TRH, which has been observed in rats, indicating that a decrease in TRH may prompt a reduction in acetylcholine, which plays a significant role in the development of AD (32, 33).

The strengths of the present study involved the numerous participants who were recruited from a representative, nationwide population sample and the detailed information regarding the various covariates adjusted as confounding factors in the analyses. Thus, we were able to adjust for plausible confounding factors and perform stratified subgroup analyses to identify potentially relevant interactions. Although the diagnosis of AD was based on ICD-10 coding, the coding of AD from NHIS data had good accuracy and validity, as described in our previous literature (18, see **File S2**). We calculated the interaction for each relationship between the independent variables and basic demographics including age, sex, income, and region of residence in model 2 (**Table S3**). We confirmed that there was little interaction between them in these analyses. The last strength of the present study is that this work simultaneously addresses hypothyroidism and hyperthyroidism in a single study and includes adjustment for previously undisclosed factors, such as each thyroid disease and thyroid medication (levothyroxine). Nevertheless, there are some limitations to our analysis that warrant consideration. First, due to the cross-sectional character of the study, our analysis could not conclude whether thyroid diseases influenced and contributed to the development or progression of AD or whether they were a result of AD. In fact, several studies have proposed that altered thyroid hormone levels can be a consequence of AD. One study explained that adenohypophysis deterioration due to degenerative changes in the AD brain could lead to a decrease in TRH and TSH production, resulting in low thyroid hormone levels (13). Moreover, another study reported that hypofunction of the adenohypophysis due to circadian rhythm disturbances in AD decreased TSH levels (34). Although it is not elusive whether thyroid dysregulation occurs before or after the onset of AD, treatment with thyroid hormone restoration has been reported to potentially improve cognitive deficits in animal studies (35–37). We performed an additional analysis excluding individuals who were diagnosed with thyroid disease 2 years before the index date to compensate for the characteristics of this study, which had a case-control design (**Table S4**). Future prospective studies are essential to confirm and clarify the causal association between thyroid disease and AD. The second limitation is that we cannot rule out the effect of some unconsidered confounders. Although we adjusted our risk estimate analyses for various comorbid diseases and medical conditions, in particular cardiovascular risk factors, to minimize the effect of comorbidity, other brain pathologies related to thyroid dysfunction may remain. In addition to cardiovascular disease, thyroid hormone dysregulation has been implicated in or is comorbid with hippocampal sclerosis, autoimmune diseases, and cerebrovascular disease, such as cerebral small vessel disease and stroke (38–41). Additional research controlling for these brain pathologies influencing thyroid function is required to elucidate the contribution of thyroid disease to AD. Furthermore, other putative risk factors for AD, including genetic risk factors and well-established acquired factors, such as stress, depression, physical activity, occupation, and hearing loss, were not considered in this study (42). Further analyses after adjusting for putative confounders are required to clarify these associations. Third, since information about serum thyroid hormone levels and thyroid gland function were not available in the claims database, we could not consider them in this analysis. Thus, we could not determine to what extent the degree of thyroid function abnormalities affected AD in the study. Fourth, we could not rule out the possibility of overlapping participants with thyroid diseases with those who had taken levothyroxine. To compensate for this limitation, each exposure, such as thyroid diseases or conditions, was additionally adjusted in model 2. The final limitation is that the effect size of the associations of thyroid diseases and AD was relatively small. Therefore, we calculated the required number to harm for AD in order to show an absolute effect (**Table S5**).

Although we could not confirm a causal relationship due to the cross-sectional design, our results imply that efforts to properly manage thyroid diseases may be beneficial in the prevention of AD.

## Conclusion

The current results demonstrated that clinical hypothyroidism, thyroiditis, and hyperthyroidism were significantly associated with AD, although the biological mechanisms remain unclear. Furthermore, our findings imply that there may be a role for the treatment of these thyroid diseases in prevention of the development or progression of AD. Further research is warranted to elucidate causality and the directions of these associations.

## Data Availability Statement

The data analyzed in this study is subject to the following licenses/restrictions: The current article used a national sample cohort and does not involve data that can be available. Requests to access these datasets should be directed to https://nhiss.nhis.or.kr/bd/ay/bdaya001iv.do.

## Ethics Statement

This study was approved by the ethics committee of Hallym University (2020-07-022). Written informed consent for participation was not required for this study in accordance with the national legislation and the institutional requirements.

## Author Contributions

JK and HL participated in the interpretation of the data and drafted and revised the manuscript. YK, MK, J-HK, CM, and DY participated in data collection and data interpretation. HC designed the study, participated in data collection and data interpretation, and revised the manuscript. All authors contributed to the article and approved the submitted version.

## Funding

This work was supported in part by a research grant (NRF-2021R1C1C1004986) from the National Research Foundation (NRF) of Korea.

## Conflict of Interest

The authors declare that the research was conducted in the absence of any commercial or financial relationships that could be construed as a potential conflict of interest.

## Publisher’s Note

All claims expressed in this article are solely those of the authors and do not necessarily represent those of their affiliated organizations, or those of the publisher, the editors and the reviewers. Any product that may be evaluated in this article, or claim that may be made by its manufacturer, is not guaranteed or endorsed by the publisher.
