# Supplementary material for: The Association Between Thyroid Diseases and Alzheimer’s Disease in a National Health Screening Cohort in Korea
Source: Front Endocrinol (Lausanne). 2022 Mar 7;13:815063. doi: 10.3389/fendo.2022.815063 (PMC8936176; doi:10.3389/fendo.2022.815063)
Supplement: Supplementary file 1 [file Table_1.docx]

**Table S1** Pearson’s chi-square test between levothyroxine treatment, goiter, hypothyroidism, thyroiditis, and hyperthyroidism.

|  |  | **Levothyroxine treatment** | **Goiter** | **Hypothyroidism** | **Thyroiditis** | **Hyperthyroidism** |
| --- | --- | --- | --- | --- | --- | --- |
| Levothyroxine treatment | χ² | 1 |  |  |  |  |
|  | P-value |  |  |  |  |  |
| Goiter | χ² | 10683.43 | 1 |  |  |  |
|  | P-value | <0.001^*^ |  |  |  |  |
| Hypothyroidism | χ² | 34771.16 | 4398.96 | 1 |  |  |
|  | P-value | <0.001^*^ | <0.001^*^ |  |  |  |
| Thyroiditis | χ² | 3483.52 | 1808.35 | 2457.95 | 1 |  |
|  | P-value | <0.001^*^ | <0.001^*^ | <0.001^*^ |  |  |
| Hyperthyroidism | χ² | 3283.56 | 1320.91 | 2371.23 | 785.36 | 1 |
|  | P-value | <0.001^*^ | <0.001^*^ | <0.001^*^ | <0.001^*^ |  |

^*^Chi-square test. Significance at P <0.05.
