# Supplementary material for: The Association Between Thyroid Diseases and Alzheimer’s Disease in a National Health Screening Cohort in Korea
Source: Front Endocrinol (Lausanne). 2022 Mar 7;13:815063. doi: 10.3389/fendo.2022.815063 (PMC8936176; doi:10.3389/fendo.2022.815063)
Supplement: Supplementary file 2 [file Table_2.docx]

**FILE S2** Description of the diagnosis of dementia.

Dementia was categorized if the participants were diagnosed Alzheimer's disease (G30) or Dementia in Alzheimer's disease (F00). We selected if the participants were treated ≥2 times.

In this national sample cohort, 123,025 participants were ≥65 years old in 2012 year. Among them, 9,740 (7.9%) of participants were categorized as dementia according to our methods (5.4% [n =2,758] in male; 9.7% [n= 6,982] in female).

We could compare these results of central dementia center of Korea ([www.nid.or.kr](http://www.nid.or.kr)) which is controlled by Ministry of Health and Welfare of Korea. The earliest data was 2012 year, and it was available in ≥65 years old. According to their data, the prevalence of dementia (Alzheimer's disease and others) except vascular dementia were 7.63% (4.47% in male; 9.85% in female).
