# Supplementary material for: The Association Between Thyroid Diseases and Alzheimer’s Disease in a National Health Screening Cohort in Korea
Source: Front Endocrinol (Lausanne). 2022 Mar 7;13:815063. doi: 10.3389/fendo.2022.815063 (PMC8936176; doi:10.3389/fendo.2022.815063)
Supplement: Supplementary file 3 [file Table_3.docx]

**TABLE S3** The P-value of each interaction analysis between independent variables and basic demographics (age, sex, income, region of residence) in Model 2.

|  | **Age** | **Sex** | **Income** | **Region of residence** |
| --- | --- | --- | --- | --- |
| Levothyroxine treatment | 0.317 | 0.531 | 0.737 | 0.908 |
| Goiter | 0.045^*^ | 0.288 | 0.859 | 0.864 |
| Hypothyroidism | 0.780 | 0.310 | 0.270 | 0.685 |
| Thyroiditis | 0.554 | 0.066 | 0.412 | 0.856 |
| Hyperthyroidism | 0.738 | 0.093 | 0.163 | 0.982 |

^*^Significance at P <0.05.
