# Supplementary material for: The Association Between Thyroid Diseases and Alzheimer’s Disease in a National Health Screening Cohort in Korea
Source: Front Endocrinol (Lausanne). 2022 Mar 7;13:815063. doi: 10.3389/fendo.2022.815063 (PMC8936176; doi:10.3389/fendo.2022.815063)
Supplement: Supplementary file 4 [file Table_4.docx]

**TABLE S4** Crude and adjusted odds ratios (95% confidence intervals) associated with levothyroxine treatment, goiter, hypothyroidism, thyroiditis, and hyperthyroidism in Alzheimer’s disease except for individuals diagnosed with thyroid diseases for 2 years before the index date.

| **Characteristics** | | **N of**  **Thyroid disease patients** | **N of Controls** | **Odds ratios for Alzheimer’s disease** | |
| --- | --- | --- | --- | --- | --- |
|  |  | **(exposure/total, %)** | **(exposure/total, %)** | **Adjusted model^†^** | **P-value** |
| Total participants (n= 80,772) | | | |  |  |
|  | Goiter | 428/16,065 (2.7%) | 1,624/64,707 (2.5%) | 1.13 (1.00-1.27) | 0.045^*^ |
|  | Hypothyroidism | 409/16,065 (2.5%) | 1,628/64,707 (2.5%) | 0.96 (0.83-1.12) | 0.625 |
|  | Thyroiditis | 206/16,065 (1.3%) | 696/64,707 (1.1%) | 1.19 (0.92-1.54) | 0.031^*^ |
|  | Hyperthyroidism | 308/16,065 (1.9%) | 1,071/64,707 (1.7%) | 1.14 (1.00-1.31) | 0.053 |

*CCI, Charlson comorbidity index.*

^*^Unconditional logistic regression model, Significance at P <0.05.

^†^Adjusted model was adjusted for age, sex, income, and region of residence, obesity, smoking, alcohol consumption, systolic blood pressure, diastolic blood pressure, fasting blood glucose, total cholesterol, hemoglobin, thyroid cancer, CCI score, levothyroxine treatment, goiter, hypothyroidism, thyroiditis, and hyperthyroidism.
