# Supplementary material for: The Association Between Thyroid Diseases and Alzheimer’s Disease in a National Health Screening Cohort in Korea
Source: Front Endocrinol (Lausanne). 2022 Mar 7;13:815063. doi: 10.3389/fendo.2022.815063 (PMC8936176; doi:10.3389/fendo.2022.815063)
Supplement: Supplementary file 5 [file Table_5.docx]

**TABLE S5** The Number needed to harm for Alzheimer’s disease.

| **Exposure** | | **Number needed to harm** |
| --- | --- | --- |
|  | Levothyroxine treatment | 60.84 |
|  | Goiter | 71.12 |
|  | Hypothyroidism | 41.97 |
|  | Thyroiditis | 26.72 |
|  | Hyperthyroidism | 34.10 |
